# A Dynamic View of Molecular Switch Behavior at Serotonin Receptors: Implications for Functional Selectivity

**DOI:** 10.1371/journal.pone.0109312

**Published:** 2014-10-14

**Authors:** Maria Martí-Solano, Ferran Sanz, Manuel Pastor, Jana Selent

**Affiliations:** Research Programme on Biomedical Informatics (GRIB), Department of Experimental and Health Sciences, Universitat Pompeu Fabra, IMIM (Hospital del Mar Medical Research Institute), Barcelona, Spain; Medical School of Hannover, Germany

## Abstract

Functional selectivity is a property of G protein-coupled receptors that allows them to preferentially couple to particular signaling partners upon binding of biased agonists. Publication of the X-ray crystal structure of serotonergic 5-HT_1B_ and 5-HT_2B_ receptors in complex with ergotamine, a drug capable of activating G protein coupling and β-arrestin signaling at the 5-HT_1B_ receptor but clearly favoring β-arrestin over G protein coupling at the 5-HT_2B_ subtype, has recently provided structural insight into this phenomenon. In particular, these structures highlight the importance of specific residues, also called micro-switches, for differential receptor activation. In our work, we apply classical molecular dynamics simulations and enhanced sampling approaches to analyze the behavior of these micro-switches and their impact on the stabilization of particular receptor conformational states. Our analysis shows that differences in the conformational freedom of helix 6 between both receptors could explain their different G protein-coupling capacity. In particular, as compared to the 5-HT_1B_ receptor, helix 6 movement in the 5-HT_2B_ receptor can be constrained by two different mechanisms. On the one hand, an anchoring effect of ergotamine, which shows an increased capacity to interact with the extracellular part of helices 5 and 6 and stabilize them, hinders activation of a hydrophobic connector region at the center of the receptor. On the other hand, this connector region in an inactive conformation is further stabilized by unconserved contacts extending to the intracellular part of the 5-HT_2B_ receptor, which hamper opening of the G protein binding site. This work highlights the importance of considering receptor capacity to adopt different conformational states from a dynamic perspective in order to underpin the structural basis of functional selectivity.

## Introduction

The phenomenon of functional selectivity, by which G protein-coupled receptors (GPCRs) can differentially activate particular intracellular signaling pathways upon interaction with biased agonists [1], is currently shedding light into the complex mechanisms of GPCR function, as well as unveiling new opportunities for the discovery of safer and more efficacious drugs possessing pathway selectivity [2]–[5]. This complex receptor behavior has been linked to the existence of several receptor conformational states with a different ability to couple to intracellular signal transducers [6], [7]. However, despite the increasing availability of GPCR crystal structures, the nature of these diverse receptor conformations and the structural basis of their differential interactions with ligands and intracellular signaling proteins is still unsolved. In this context, molecular dynamics (MD) simulations can complement X-ray structural data by yielding information on the stability of different ligand-receptor interactions as well as on the capability of these interactions to favor particular receptor conformational states [8].

**Figure 1:**
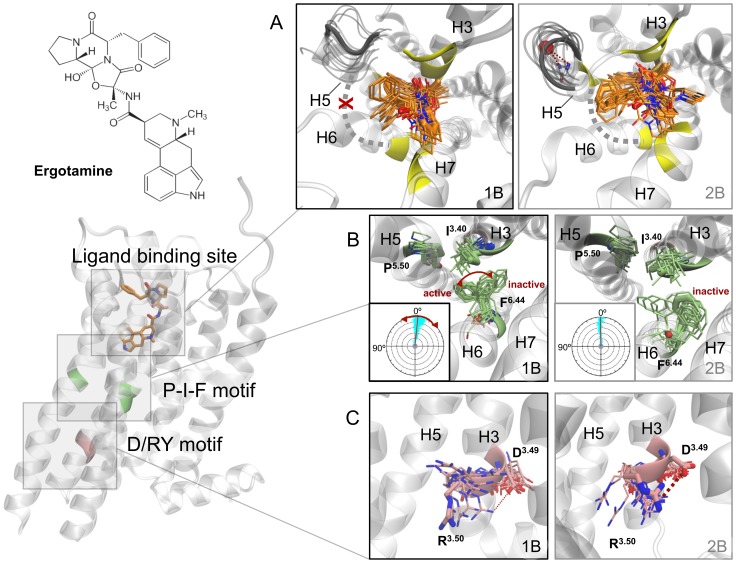
Ergotamine structure and schematic representation of the position of micro-switches in the ergotamine-serotonin receptor complexes. X-crystal structures are shown in thick sticks representation whereas simulation shows frames every 200 ns of the 2.5 µs concatenated trajectories. A) Representative ergotamine poses (depicted in orange) in its binding pocket (top ten receptor interacting residues in yellow). The extracellular part of helix 5 (grey) forms and additional turn stabilized by water (red) in the 5-HT_2B_ receptor. Extended interactions of ergotamine with helix 5 can anchor together helix 5 and 6 (grey dashed line connecting yellow interacting residues), an effect which is not seen in the 5-HT_1B_ receptor (red cross) B) Relative positions of F^6.44^ of the P-I-F motif in both receptors and amount of helix 6 rotation (from residue 6.44 to 6.50) measured with the SIMULAID framework for the analysis of MD simulations (inset). C) Different conformations of the residues forming the D/RY motif in the 5-HT_1B_ and 5-HT_2B_ receptors.

In the present study, we have applied all-atom MD simulations, covering a simulation time of more than 6 µs, to analyze the structural basis of biased signaling at serotonin receptors. For this purpose, we have studied two recently crystallized GPCRs: the serotonergic 5-HT_1B_ and 5-HT_2B_ receptors (5-HT_1B_R and 5-HT_2B_R, PDB IDs: 4IAR and 4IB4) [9], [10]. These receptors have been crystallized in complex with ergotamine, an anti-migraine drug which can activate to similar extents G_i_ protein and β-arrestin coupling at the 5-HT_1B_R but favors β-arrestin over G_q_ protein coupling at the 5-HT_2B_R. Understanding selectivity at these receptors is of special interest as agonism at the 5-HT_1B_R has been associated to the anti-migraine effect of ergotamine while agonism at the 5-HT_2B_R seems to be related to undesired valvulopathic effects [11], [12]. In their accompanying publications, the authors analyze the particular conformations of a series of residues - known as molecular micro-switches [13] - which are supposed to determine the nature of receptor activation. In particular, they relate the different receptor states observed in the crystals to a particular combination of activation states of these micro-switches. In the 5-HT_1B_R, they associate increased G protein coupling to a higher degree of activation of helix VI, which can be linked to an active state of the P-I-F motif at the base of the ligand binding pocket and also of the D(E)/RY motif in the intracellular opening of the receptor (Figure 1). In the 5-HT_2B_R, these two switches are in their intermediate or inactive state but, conversely, helix VII seems to be in an active-like state as exemplified by the conformation of Y^7.53^ of the NPxxY motif, an observation which the authors link to higher β-arrestin coupling capability.

However, the analysis of the static picture that crystal structures provide makes it difficult to understand how information on the different states of individual switches is transmitted across the receptors; in particular, if we consider the relative spatial separation between the different switches, as well as their distance to the ligand orthosteric binding pocket. Fortunately, the progress made in accelerated molecular dynamics simulations [14] is nowadays helping to gain insight into different aspects of GPCR function such as receptor activation-inactivation processes [15], stabilization of different receptor populations by agonists and inverse agonists [16] or even ligand binding [17], [18]. In our case, this technique allows obtaining a dynamic view of the molecular basis of ergotamine-dependent signaling at the 5-HT_1B_R and 5-HT_2B_R.

## Results

In order to evaluate the behavior of both receptors, we have run unbiased MD simulations of 5-HT_1B_R and 5-HT_2B_R consisting of 5 independent replicates per system of 500 ns each (for further details please refer to Table 1 and to the Methods Section).

**Table 1:** Details of the molecular dynamics simulations performed.

Simulation description	Length	Replicates
5-HT_1B_ and 5-HT_2B_R with ergotamine	500 ns	5*
5-HT_1B_ and 5-HT_2B_R apo form	500 ns	1*
5-HT_2B_R F^6.41^L mutant	500 ns	1
5-HT_1B_ and 5-HT_2B_R with LSD	500 ns	1*
5-HT_2B_R metadynamics	200 ns	6
Total simulation time	8.7 µs	

*This number of replicates was performed for each receptor.

### Ergotamine Interactions

Our results show that the positions of ergotamine in the binding pocket of both receptors are stable over the simulated time (with an average RMSD of 1.98 Å for the 5-HT_1B_ receptor and 1.94 Å for the 5-HT_2B_ receptor) and consistent with the ones presented in the recently published crystal structures. Thus, ergotamine adopts a similar position in the orthosteric binding pocket of both receptors (Figure 1A, see Figure S1 for a detailed representation), but establishes increased contacts with an extended binding pocket in the extracellular part of helix 5 of the 5-HT_2B_R, which are not present in the 5-HT_1B_R (depicted in yellow in Figure 1A). As a consequence, in the 5-HT_1B_R, water molecules can enter from the extracellular side into the binding pocket occupying a space between ergotamine and helix 5; a water entrance that is not seen in the 5-HT_2B_R (see water occupancy map, Figure S2). Interestingly, the capability of ergotamine to interact with helix 5 to a higher degree in the crystal structure of 5-HT_2B_R has been related to the presence of an additional helical turn in the extracellular part of this helix, which is absent in the 5-HT_1B_ receptor (highlighted in dark grey in Figure 1A) [10]. Such difference in conformation also seems to be related to the presence of a water molecule in the crystal structure (Figure 1A (right) and Figure S3), which interacts with residues E212^5.33^, D216^5.37^ and G215^5.36^ of the 5-HT_2B_R (according to Ballesteros-Weinstein residue numbering [19]). In fact, monitoring water molecules near position 5.36 in our dynamic systems shows a constant interaction with a water molecule in the 5-HT_2B_R (with an average water content along the simulation of 1.4 within 2 Å of the Cα of G^5.36^), which is not observed in the 5-HT_1B_R. Further assessment of the conformational stability of this region points to a stable turn in the 5-HT_2B_R, which is maintained even in the absence of ergotamine for 500 ns, and which is not present in the 5-HT_1B_ subtype (see dark gray region in Figure 1A and Figure S4 for a time series analysis of its secondary structure).

### Differential Molecular Switch Stability

After corroborating the stability of the differential ergotamine/receptor interactions observed in the crystal structures, we assessed the behavior of the proposed molecular micro-switches, which have been linked to differential G protein coupling. One of these switches is the so-called P-I-F motif. This switch - which is formed by residues P^5.55^, I^3.40^ and F^6.44^ - seems to be in different activation states in the two serotonin receptor crystal structures. Specifically, in the 5-HT_1B_R, F^6.44^ adopts a conformation facing helix 5 (Figure 1B (left) active F^6.44^ state), which is close to the one found in the active state of the β2-adrenergic crystal structure in complex with a G_s_ protein (PDB ID: 3SN6 [20]). In contrast, in the 5-HT_2B_R, this residue is located nearer to helix 7 (Figure 1B (right) inactive F^6.44^ state) and, therefore, is closer to the position of F^6.44^ in the carazolol-bound inactive form of the β2-adrenergic receptor (PDB ID: 2RH1 [21]). Importantly, monitoring the dynamic properties of these switches revealed an interesting behavior that is not deducible from the static picture that X-ray structures provide. In detail, while in the 5-HT_2B_R the inactive conformation of F^6.44^ prevails, in the 5-HT_1B_R there is a higher transition between active/inactive states of this residue with a preference for the active state (Table 2, Figure 1B). This active state preference seems to be related to an increased capacity of the upper part of helix 6 to rotate visiting its active and its inactive states by spanning a 12° region (inset, Figure 1B and Figure S4). The degree of rotation seems to be modulated by contacts of ergotamine with both helix 5 and 6 in the 5-HT_2B_R (Figure 1A, grey dashed line and Figure S1). These contacts, which are not present in the 5-HT_1B_R, contribute to an anchoring of helix 6 in the 5-HT_2B_ receptor which shows a maximal rotation of around 5° (inset, Figure 1B and Figure S5). This behavior is also consistent with the hypothesis by Wacker *et al.* linking a higher degree of interaction of ergotamine with helix 5 in the 5-HT_2B_ with a lower degree of mobility of helix 6 which, in turn, would hamper receptor conformational changes related to G protein activation [10].

**Table 2:** Percentage of engagement of proposed molecular switches in both receptors across MD replicates.

Molecular switch	5-HT_1B_ simulations^[a]^	5-HT_2B_ simulations^[a]^
F^6.44^ activation ^[b]^	54%	32%
D^3.49^-R^3.50^ salt bridge formation	38%	77%
Ionic lock closure	14%	73%

[a] Calculated over 2.5 ìs simulation time per system.

[b] Measured as distance towards helix 5 (see Figure 3).

**Figure 2:**
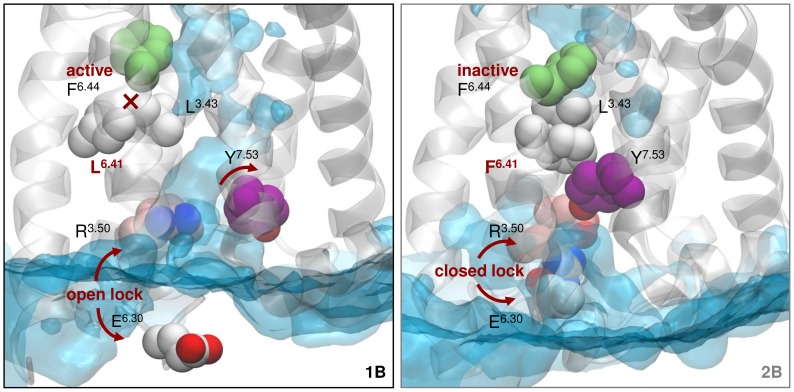
Hydrophobic connector region formed by positions 3.43, 6.41 and 6.44 between helices 3 and 6. In this figure, we see that in the 5-HT_1B_R (left) the P-I-F motif adopts an active conformation and the ionic lock remains open, while in the 5-HT_2B_R (right), these switches are preferentially in an inactivated state. This can be related to the fact that in the 5-HT_2B_R, the inactive conformation of F^6.44^ in the P-I-F motif is stabilized by hydrophobic interactions with F^6.41^ and L^3.43^. Stronger contacts between helices 3 and 6 favor the formation of the ionic lock (R^3.50^-E^6.30^) in this receptor. In contrast, these interactions are mainly lost in the 5-HT_1B_R (red cross) due to sequence differences. In line with this observation, analysis of water occupancy at bulk concentration shows that in the 5-HT_2B_ receptor the hydrophobic interactions between helix 3 and 6 hamper the formation of a water channel (in blue) present in the 5-HT_1B_ receptor. That seems to impact the position of residue 7.53 (purple) which is stable in the 5-HT_2B_ receptor but moves towards helix 2 in the 5-HT_1B_R (red arrow).

Another important micro-switch, the D(E)/RY motif, has been closely related to the capacity of receptors to couple with the C-terminus region of G proteins [20], [22]. In the 5-HT_2B_R crystal structure, R^3.50^ and D^3.49^ form a salt bridge, a characteristic interaction found in inactivated GPCRs [10]. Conversely, in the 5-HT_1B_R, this bridge is broken, potentially allowing interaction of R^3.50^ with a G protein. Noteworthily, our dynamic models (Figure 1C) show that there is not just a static interaction state between R^3.50^ and D^3.49^, but rather an equilibrium between an established and a disrupted salt bridge. Nevertheless, in agreement with the crystal structures, the calculated percentage of salt bridge formation over the total simulation for both systems shows a predominately open (active) state for the 5-HT_1B_R and a predominantly closed (inactive) state for the 5-HT_2B_R (Table 2). Besides that, the 5-HT_2B_R presents a higher tendency for inactivation, as the ionic lock formed by the interaction of residues R^3.50^ and E^6.30^, which controls receptor opening and closure at the intracellular side, tends to close, while remaining open in the 5-HT_1B_R (see Figure 2 and Table 2). As a control, we also simulated both receptors in the absence of ergotamine. As expected, we observe receptor inactivation in both apo forms (Figure S6). However, the ionic lock transits quicker to the inactivated state in the 5-HT_2B_R compared to the 5-HT_1B_R.

### Receptor Determinants of Micro-Switch Behavior

At this point, a fundamental question arose about the mechanisms of signal propagation between the distantly located ionic lock and the P-I-F motif. Analysis of the receptor region adjacent to the ionic lock and the P-I-F motif residues, led us to propose a hydrophobic connector region that mediates communication between these functionally relevant switches (Figure 2). Thus, as we can observe in Figure 2 (right), the inactive state of F^6.44^ in the P-I-F motif of the 5-HT_2B_R seems to be stabilized by hydrophobic interactions with L^3.43^ in helix 3 and with residue F^6.41^ in its same helix; which are not formed in the 5-HT_1B_R (left, red cross). Due to this stronger intramolecular helical packing between helix 3 and 6 towards the intracellular side of the 5-HT_2B_R, the receptor seems to be less prone to the intracellular opening motion of helix 6 which is necessary for G protein coupling. This is indicated by the 5-HT_2B_R tendency to preferentially adopt a closed ionic lock conformation (Table 2). Interestingly, virtual mutation of residue F^6.41^ near the P-I-F motif at the 5-HT_2B_R to a leucine, as found in the 5-HT_1B_R, results in an altered receptor behavior with an ionic lock and a P-I-F motif capable of transiting to their active states (Figure S7). Conversely, MD simulations of the 5-HT_2B_R in complex with LSD, a compound lacking the tripeptide moiety of ergotamine and promoting a lower degree of bias at this receptor [10], did not show a transition to an activated state of the ionic lock and the P-I-F motif (Figure S8). These results would point to a higher impact of sequence differences on receptor activation propensity as opposed to differences in ligand binding. However, we should consider that mutation of position 6.41 could have faster effects on receptor activation than differences in ligand-receptor interactions (LSD vs. ergotamine) due to their different proximity to the studied molecular switches. In this regard, future availability of receptor crystals containing LSD would allow clarifying the current picture.

**Figure 3:**
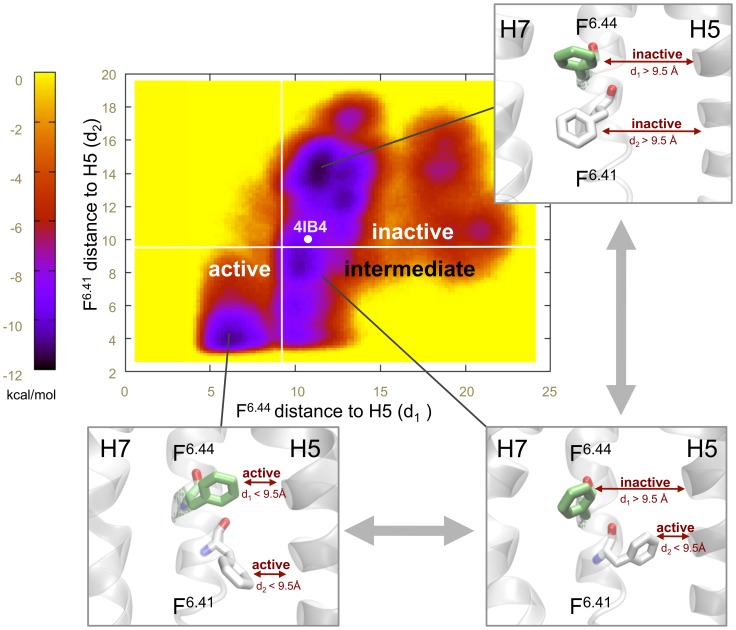
Two-dimensional free energy profile of the connector region of the 5-HT_2B_R. To create this map two collective variables were used: the first coordinate (d_1_) is the distance between C-4 of the F^6.44^ and the Cα of residues 5.50, while the second one (d_2_) is the distance between the C-4 of F^6.41^ and the Cα of residue 5.54. As we can see, an active state of F^6.44^ only exists when residue F^6.41^ has adopted an active conformation and broken their hydrophobic contact. The point 4IB4 represents distances as they are found in the crystal structure of the 5-HT_2B_R.

In parallel to the virtual mutagenesis studies, an additional proof on the importance of hydrophobic interactions in the (in)activation of the connector region of the 5-HT_2B_R, came from enhanced MD sampling using a metadynamics approach. In particular, we assessed the impact of the conformation of F^6.41^ on the activation state of F^6.44^ in the P-I-F motif. As a measure of the activation state, we used the distance to helix 5 of residues F^6.44^ and F^6.41^ (d_1_ and d_2_) as collective variables (CVs) (Figure 3, structural insets). This distance was selected after analyzing the P-I-F motif in the aforementioned β2-adrenergic receptor X-ray crystal structures in their activated and inactivate state, which led us to define the following activation threshold: inactive state >9.5 Å≥ active state. Interestingly, the two-dimensional free energy profile obtained from metadynamics (total simulation time: 1.2 µs) revealed an (in)activation pathway of residue F^6.44^ in the P-I-F motif that involves a concerted motion with residue F^6.41^ (Figure 3). In detail, starting from the inactive structure (d_1_ and d_2_>9.5 Å), F^6.41^ undergoes a conformational change approaching helix 5 (d_2_<9.5 Å, see intermediate state in Figure 3), thus disrupting stabilizing hydrophobic interactions between F^6.41^ and F^6.44^. This intermediate state seems to allow a conformational change of F^6.44^ towards its active state (d_1_<9.5 Å, see active state in Figure 3). Noteworthily, according to this energetic map, F^6.44^ cannot transit to its active conformation (d_1_<9.5 Å) unless F^6.41^ has already adopted an active conformation itself (d_2_<9.5 Å).

Interestingly, the importance of the presence of this hydrophobic connector is also reflected by the analysis of water entrance to the intracellular part of both receptors. As we can see in Figure 2, hydrophobic interactions between helix 3 and 6 hamper the entrance of water to the G protein binding site of the 5-HT_2B_R. Conversely, in the 5-HT_1B_R, the increased degree of receptor opening allows the formation of a water channel. The different degree of water entrance to both receptors, in turn, can help explain the position adopted by Y^7.53^ of the NPxxY motif (a switch which Wacker *et al.* associate with β-arrestin coupling) [10]. In our simulations, Y^7.53^ preserves its crystal structure position at the 5-HT_2B_R and faces water at the intracellular receptor opening (Figure 2, right). However, upon formation of the water channel in the 5-HT_1B_R, Y^7.53^ adopts a conformation facing helix 2 (see red arrow in Figure 2 left). Accordingly, analysis of distance between Y^7.53^ and helices 2 and 3 during the total simulation time (Figure S9) allows seeing how in the 5-HT_1B_R this residue abandons the initial state present in the crystal structure and tends to occupy a region closer to helix 2 and more distant to helix 3, thus separating from the center of the helix bundle. In the 5-HT_2B_R, conversely, Y^7.53^ movement is more restrained and this residue adopts a position away from helix 2 and closer to helix 3, thus maintaining an active conformation as described in the crystal structure publication. Notably, stabilization of Y^7.53^ in a position near helix 2 as the one observed for the 5-HT_1B_R has previously been described in simulations of other GPCRs, and could correspond to an intermediate residue state previous to G protein coupling [23], [24].

## Discussion

In summary, extended MD simulations can add highly relevant information to the recently obtained X-ray structures of the 5-HT_1B_ and 5-HT_2B_ receptors. Results presented in this work allow us to draw a picture on the structural basis of the different functional states of serotonin receptors. These differences are related to both the effect of ergotamine interactions with the receptors, as well as with differences in sequence conservation between the 5-HT_1B_ and 5-HT_2B_R. In the first place, differential interactions of ergotamine with the top of helices 5 and 6 (Figure 1A) determine the rotational freedom of helix 6 (Figure 1B) that, in turn, impacts the orientation and conformational properties of the P-I-F motif (Figure 2B). Interestingly, the importance of ligand interactions with an extended binding site on modulation of receptor signaling has also been experimentally observed in other GPCRs, as in the case of allosteric modulation of muscarinic M_2_ receptors [25], [26].

Besides that, our results suggest that an unconserved hydrophobic connector region between the P-I-F motif and the ionic lock (Figure 2) could be a key structural element differently impacting the formation of a water channel. This, in turn, would relate to the degree of receptor opening (disrupted ionic lock), which determines G protein coupling. The differential tendency of the G protein binding site to open can help explaining why G protein coupling is stronger in the 5-HT_1B_R and lower in the 5-HT_2B_R, whereas beta-arrestin can be activated by both receptors, resulting in functional selectivity. All in all, our results provide new mechanistic insight into the basis of the connection between ligand binding, conserved micro-switches and G protein coupling from a structural perspective and are in accordance with previously proposed GPCR activation mechanisms [15], [27], [28]. In this way, we have shown how MD simulations can complement experimental structural determinations by shedding light into the subtle crosstalk between receptor regions which, in the case of GPCR functional selectivity, ultimately determine differences in receptor signaling. This information, however, will clearly benefit from additional information coming from mutagenesis studies and from the structural analysis of these receptors in their G protein-coupled state and, naturally, also in complex with β-arrestin. This structural data will shed light into remaining questions on receptor activation such as the importance of the NPxxY motif for β-arrestin coupling. This type of information, together with a dynamic perspective as the one presented in this work, can provide a starting point for the rational selection of compounds engaging particular micro-switches to different extents and, in this way, favoring a particular intracellular activity pattern.

## Methods

### System preparation

5-HT_1B_ and 5-HT_2B_ receptors (PDB IDs 4IAR [9] and 4IB4 [10]) were retrieved from the Protein Data Bank. Residues were assigned numbers according to the numbering scheme proposed by Ballesteros and Weinstein [19] and the region of the crystal corresponding to the fusion protein BRIL was removed for the subsequent simulations. The protonation state of titratable groups was predicted for a pH value at 7.4 based on PROPKA [29] using the implemented prediction tool of the MOE package [30]. Subsequently, in order to place both receptors into the bilayer membrane, a hole was generated in a pre-equilibrated palmitoyloleoylphosphatidylcholine (POPC) bilayer – generated using the CHARMM-GUI Membrane Builder [31] – by removing POPC molecules. Lipids which were in close contact with the protein atoms (<1 Å distance from any protein atoms) were deleted. Finally, the coordinates for water and ions were generated using the solvate and autoionize modules of VMD 1.9.1 [32]. The ionic strength was kept at 0.15 M by NaCl and we used the TIP3 water model. The all-atom models of each system were generated by using the Amber99SB force-field parameters and ergotamine, LSD and POPC were parameterized using Antechamber from AmberTools 11 [33]. To obtain the systems of LSD in complex with both 5-HT receptors, we simply modified ergotamine to create LSD. This involved deletion of N-substitution of the lysergamide of ergotamine and subsequent addition of two ethyl groups yielding N,N-diethyl-lysergamide (LSD). The system was parameterized as mentioned above.

### Molecular dynamics simulations

Simulations were performed using ACEMD [14], [34] using the following protocol: In a first stage, each system was submitted to a minimization procedure for 3000 steps. In a second stage, the system was equilibrated using the NPT ensemble with a target pressure equal to 1.01325 bar, a time-step of 2 fs and using the RATTLE algorithm for the hydrogen atoms. In this stage, the harmonic constraints applied to the heavy atoms of the protein and ligand were progressively reduced from an initial value of 10 kcal/mol/Å until an elastic constant force equal to 0 kcal/mol and the temperature was increased to 300 K. The purpose of this relaxation phase is to allow for a complete adjustment of membrane lipids to the receptor, thus filling non-physiological gaps between receptor and membrane lipids. All the simulations were conducted using the same non-bonded interaction parameters, with a cutoff of 9 Å, a smooth switching function of 7.5 Å and the non-bonded pair list set to 9 Å. The periodic boundary conditions were set to a size of 78×78×88, and for the long range electrostatics we used the PME methodology with a grid spacing of 1 Å. In a third stage, production phases were performed using the NVT ensemble with aforementioned parameters but a time-step of 4 fs, and a hydrogen scaling factor of 4 (please see simulation data in File S1). This timestep is possible due to the implementation of the hydrogen mass repartitioning scheme in the ACEMD code [35]. Simulations were performed for 500 ns for individually generated starting structures (by performing stages 1 to 3). Importantly, individually-generated starting structures allow a more robust statistical analysis and thus the detection of relevant dynamic events that are independent from the starting structures.

### Metadynamics simulations

In order to enhance the sampling of the hydrophobic connector region including the residues F^6.41^ and F^6.44^, we applied a metadynamics approach as implemented in the PLUMED software plugin [36]. In order to describe the behavior of the hydrophobic connector region, we chose a two-dimensional reaction coordinate: the first dimension (d_1_) is the distance between C-4 of the F^6.44^ and the Cα of residues 5.50, while the second one (d_2_) is the distance between the C-4 of F^6.41^ and the Cα of residue 5.54 (see Figure 3). Metadynamics runs were executed using a Gaussian hill height of 0.1 kcal/mol and hill widths of 0.1 Å along both d_1_ and d_2_. The deposition rate was one hill every 4 ps, with a well-tempered bias factor of 10 [37]. To obtain a well-sampled free-energy surface, we used the multiple walker approach [38], in which several simulations explore the same free-energy landscape and interact by contributing to the same history-dependent bias potential every 20 ps. For this purpose, we used 6 walkers starting from the equilibrated receptor structure which was used for the MD production run. The system was simulated for a total of 1.2 µs using 6 walkers (200 ns each). General simulation parameters were kept as described for the production run in the previous section (please see simulation data in File S2).

### Data Analysis

Molecular images were produced using VMD 1.9.1 [32] and statistical analysis and plots were obtained using the R software [39]. Activation of molecular switches was measured as follows: i) F^6.44^ and F^6.41^ activation was measured by monitoring distance to helix 5 (measured, respectively as distance between C-4 of F^6.44^ and Cα of residue 5.50 and distance between C-4 of F^6.41^ and Cα of residue 5.54); residues were considered active when distance to H5 was equal or lower than 9.5 Å ii) ionic lock opening was measured by monitoring distance between Cε of R^3.50^ and of Cδ of E^6.30^; the lock was considered closed when this distance was equal or lower than 5 Å iii) Y^7.53^ orientation was measured by determining the distance from its OH group to the Cα of residues 3.50 and 2.40. Furthermore, assessment of helix 5 helicity in the extracellular region was evaluated using the secondary structure predictor of the Timeline plugin of VMD. The free-energy surface plot was generated using GNUPLOT [40]. Finally, helix 6 rotation measures and representations were obtained using the Trajelix module [41] of the SIMULAID framework for the analysis of molecular dynamics simulations [42].

### Supporting Information

Additional figures (S1 to S9) as well as protocols and topologies used to run molecular dynamics simulations can be found in the Supporting Information.

## Supporting Information

Figure S1 **Detailed representation of ergotamine in the binding pocket of the 5-HT_1B_ and 5-HT_2B_ receptors.** Analysis of the highest interacting residues across the replicates (using a cutoff distance of 3 Å to the ligand) yields ergotamine interactions corresponding to the ones described by Wacker *et al.* [10] Notably, differential contacts between the ligand and both receptors (depicted in purple) reveal a slightly deeper binding of ergotamine at the 5-HT_1B_R, reflected by higher contacts with residue W^6.48^, and an increased contact of the ligand with helix 5 in the 5-HT_2B_R, seen in the interaction with M^5.39^. The frequency of contacts between ergotamine and the different receptor residues depicted in the binding pocket representation can be quantitatively assessed in the bar plots at the lower part of this figure (please note that purple residues also correspond to differential ligand-receptor contacts).
(TIFF)

Figure S2 **Analysis of water occupancy in the extracellular receptor region.** Analysis of water occupancy at both receptors reflects the different space occupied by ergotamine in its extended binding pocket. While in the 5-HT_1B_R there is enough space between the ligand and helix 5 to allow water entrance, in the 5-HT_2B_R, ergotamine is closer to helix 5 and we see an increased water entrance at the level of helix 3.
(TIFF)

Figure S3 **Water-receptor interactions at the 5-HT_2B_R.** Detail of a representative snapshot showing stabilizing interactions between a water molecule and the extracellular part of helix 5.
(TIFF)

Figure S4 **Degree of helix 5 helicity at its extracellular region.** This was assessed using the VMD Timeline plugin over the total simulation time of each receptor (2.5 µs). As we can observe, while in the 5-HT_1B_R this region maintains a coiled secondary structure, in the 5-HT_2B_R the equivalent residues can adopt a turn conformation corresponding either to an α-helix or to a 3–10 helix. Secondary structure assignment via VMD of the original crystal conformations is provided below each of the plots.
(TIFF)

Figure S5 **Comparison of the amount of helix 6 rotation considering the extracellular half of the 5-HT_1B_ and 5-HT_2B_ receptors.** The amount of helix 6 rotation ranging from position 6.44 to position 6.60 was measured using the Trajelix module of the SIMULAID framework for the analysis of molecular dynamics simulations. This analysis measured rotation around the helix axis perpendicular to the membrane by considering the C alpha residues at each receptor. As we can see, helix 6 is capable of rotating to a higher degree in the 5-HT_1B_R.
(TIFF)

Figure S6 **Inactivation of the 5-HT_1B_R and the 5-HT_2B_R in the absence of ergotamine.** The upper plots monitor distance between residues forming the ionic lock (in particular of Cε of R^3.50^ and of Cδ of E^6.30^). The lower plot monitors distance of F^6.44^ to helix 5 in both receptors (dark green line, measured as distance between C-4 of this residue and Cα of residue 5.50 on H5) and distance to H5 of F^6.41^ in the 5-HT_2B_R (light green line, measured as distance between C-4 of this residue and Cα of residue 5.54 on H5). In these plots, we can see how, in both receptors, the ionic lock tends to close in the absence of ergotamine. In the 5-HT_2B_R, however, this process is much faster than in the 5-HT_1B_R. Regarding the P-I-F motif, F^6.44^ adopts an inactivated position from the beginning of the simulation, which is maintained over the 500 ns.
(TIFF)

Figure S7 **Dynamic behavior of a representative replicate of the** ***wild type*** **5-HT_2B_R and of a 5-HT_2B_R F^6.41^L mutant.** The upper plot monitors distance between residues forming the ionic lock (in particular of Cε of R^3.50^ and of Cδ of E^6.30^). The lower plot monitors distance of F^6.44^ to helix 5 (dark green line, measured as distance between C-4 of this residue and Cα of residue 5.50 on H5). In the case of the *wild type* receptor distance to H5 of F^6.41^ in the 5-HT_2B_R is also monitored (light green line, measured as distance between C-4 of this residue and Cα of residue 5.54 on H5) These plots show how, in this mutant receptor, the ionic lock and residue F^6.44^ show a bigger tendency to be in their active state.
(TIF)

Figure S8 **Dynamic behavior of the 5-HT_1B_R and 5-HT_2B_R in complex with LSD.** A) Analysis of the highest interacting residues with LSD (using a cutoff distance of 3 Å to the ligand). Purple residues correspond to differential ligand-receptor contacts while residues with an increased font represent different interactions as compared to simulations including ergotamine. B) The upper plot monitors distance between residues forming the ionic lock (in particular of Cε of R^3.50^ and of Cδ of E^6.30^). The lower plot monitors distance of F^6.44^ to helix 5 (dark green line, measured as distance between C-4 of this residue and Cα of residue 5.50 on H5). In the case of the 5-HT_2B_R the distance to H5 of F^6.41^ is also monitored (light green line, measured as distance between C-4 of this residue and Cα of residue 5.54 on H5).
(TIF)

Figure S9 **Scatterplot describing the relative distances of Y^7.53^ of the NPxxY motif to different receptor helices over the simulation replicates of both receptors.** As we can see, in the 5-HT_2B_ receptor (grey points), Y^7.53^ maintains a conformation closer to H3 (distance measured from OH of Y^7.53^ to Cα of residue 3.50). Conversely, in the 5-HT_1B_R (black points), this tyrosine adopts a position closer to H2 (reference atom Cα of residue 2.40). Red dots represent the distances observed in the crystal structures of the 5-HT_1B_R (4IAR) and 5-HT_2B_R (4IB4).
(TIFF)

File S1 **Simulation data (classical MD).** Initial topologies for the different systems simulated with classical MD (summarized in Table 1) and the scripts used to run them in ACEMD.
(TAR)

File S2 **Simulation data (metadynamics).** Initial topology of the 5-HT_2B_ receptor in complex with ergotamine, together with scripts used to perform multiple walker metadynamics.
(TAR)
