# Supplementary figures and images for: A Dynamic View of Molecular Switch Behavior at Serotonin Receptors: Implications for Functional Selectivity
Source: PLoS One. 2014 Oct 14;9(10):e109312. doi: 10.1371/journal.pone.0109312 (PMC4196896; doi:10.1371/journal.pone.0109312)

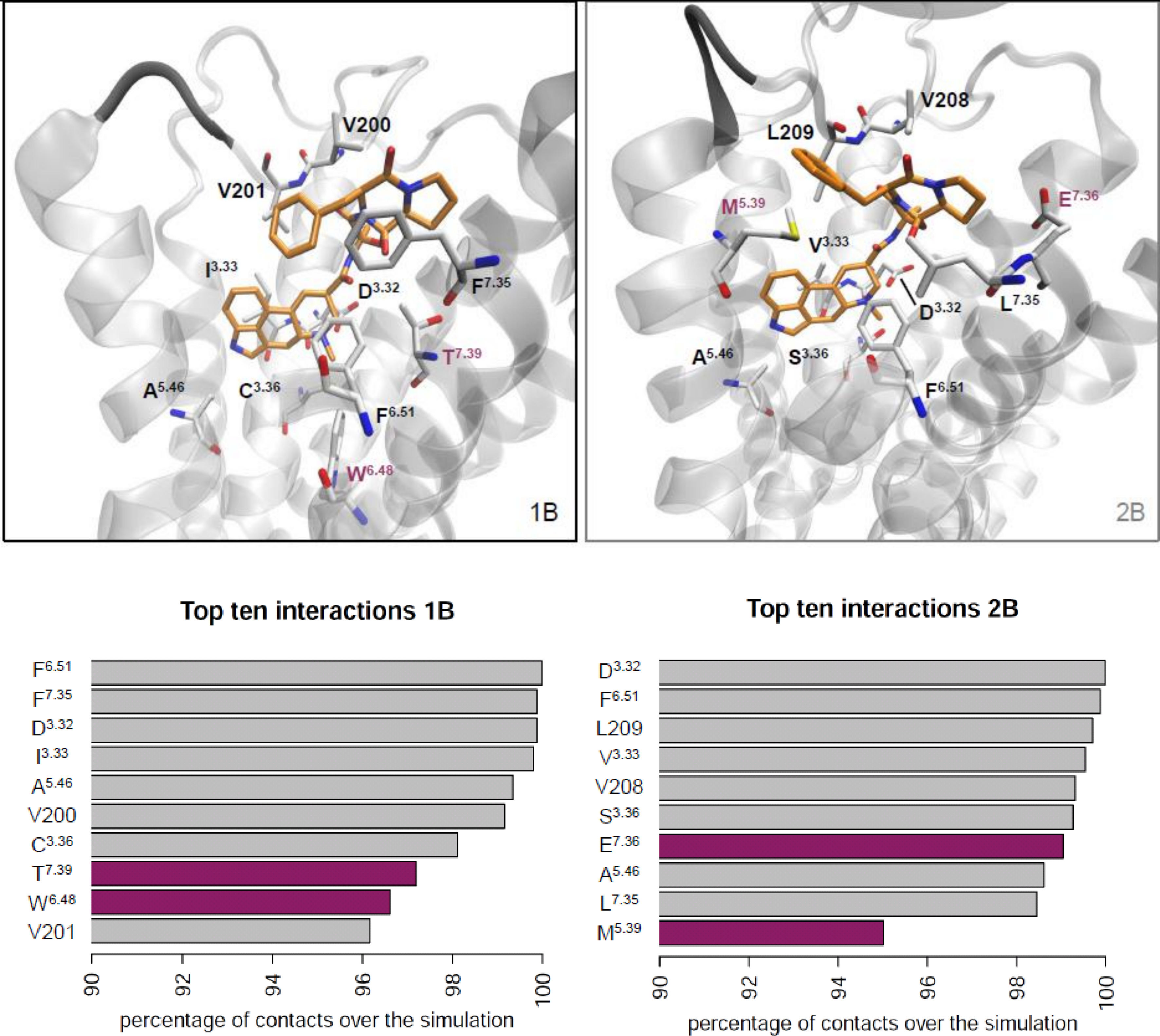

Supplement: Figure S1 — Detailed representation of ergotamine in the binding pocket of the 5-HT1B and 5-HT2B receptors. Analysis of the highest interacting residues across the replicates (using a cutoff distance of 3 Å to the ligand) yields ergotamine interactions corresponding to the ones described by Wacker et al. [10] Notably, differential contacts between the ligand and both receptors (depicted in purple) reveal a slightly deeper binding of ergotamine at the 5-HT1BR, reflected by higher contacts with residue W6.48, and an increased contact of the ligand with helix 5 in the 5-HT2BR, seen in the interaction with M5.39. The frequency of contacts between ergotamine and the different receptor residues depicted in the binding pocket representation can be quantitatively assessed in the bar plots at the lower part of this figure (please note that purple residues also correspond to differential ligand-receptor contacts). (TIFF) [file pone.0109312.s001.tiff]

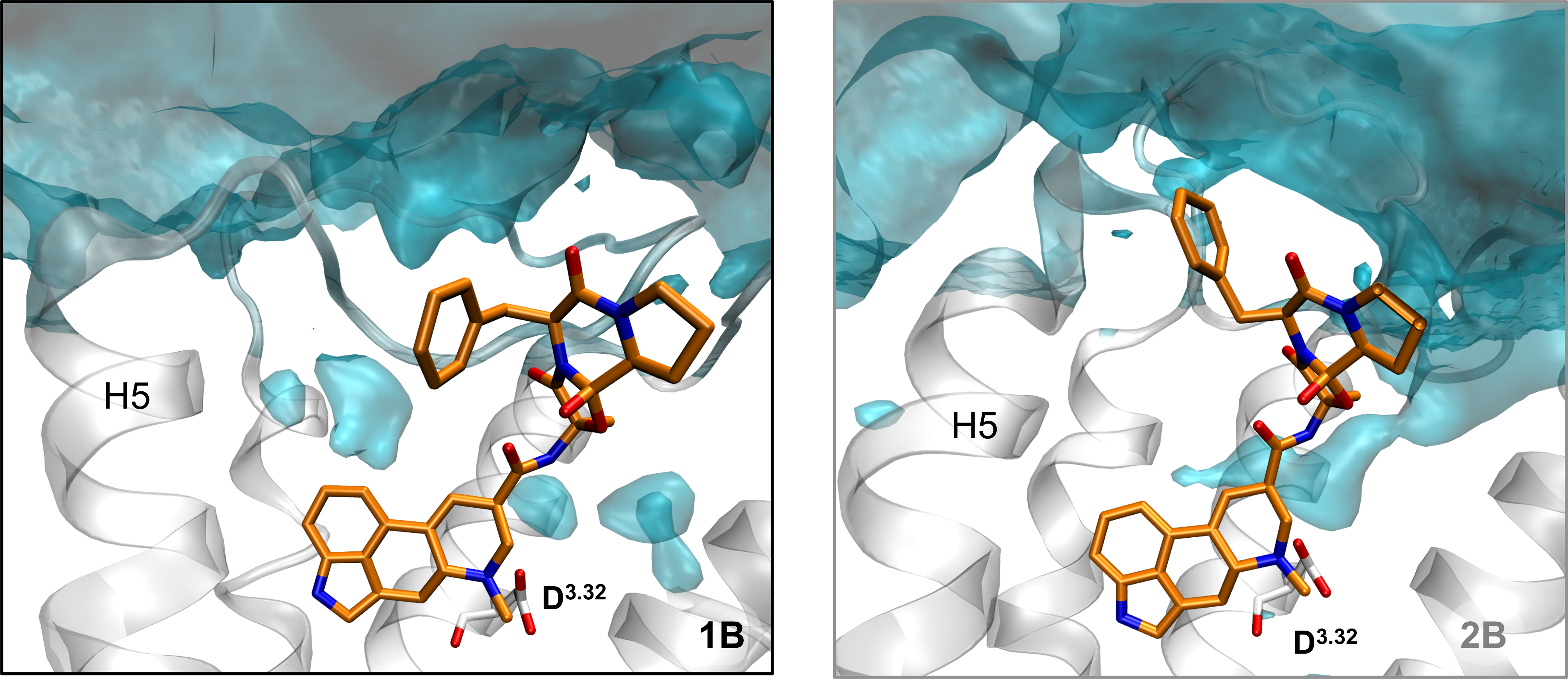

Supplement: Figure S2 — Analysis of water occupancy in the extracellular receptor region. Analysis of water occupancy at both receptors reflects the different space occupied by ergotamine in its extended binding pocket. While in the 5-HT1BR there is enough space between the ligand and helix 5 to allow water entrance, in the 5-HT2BR, ergotamine is closer to helix 5 and we see an increased water entrance at the level of helix 3. (TIFF) [file pone.0109312.s002.tiff]

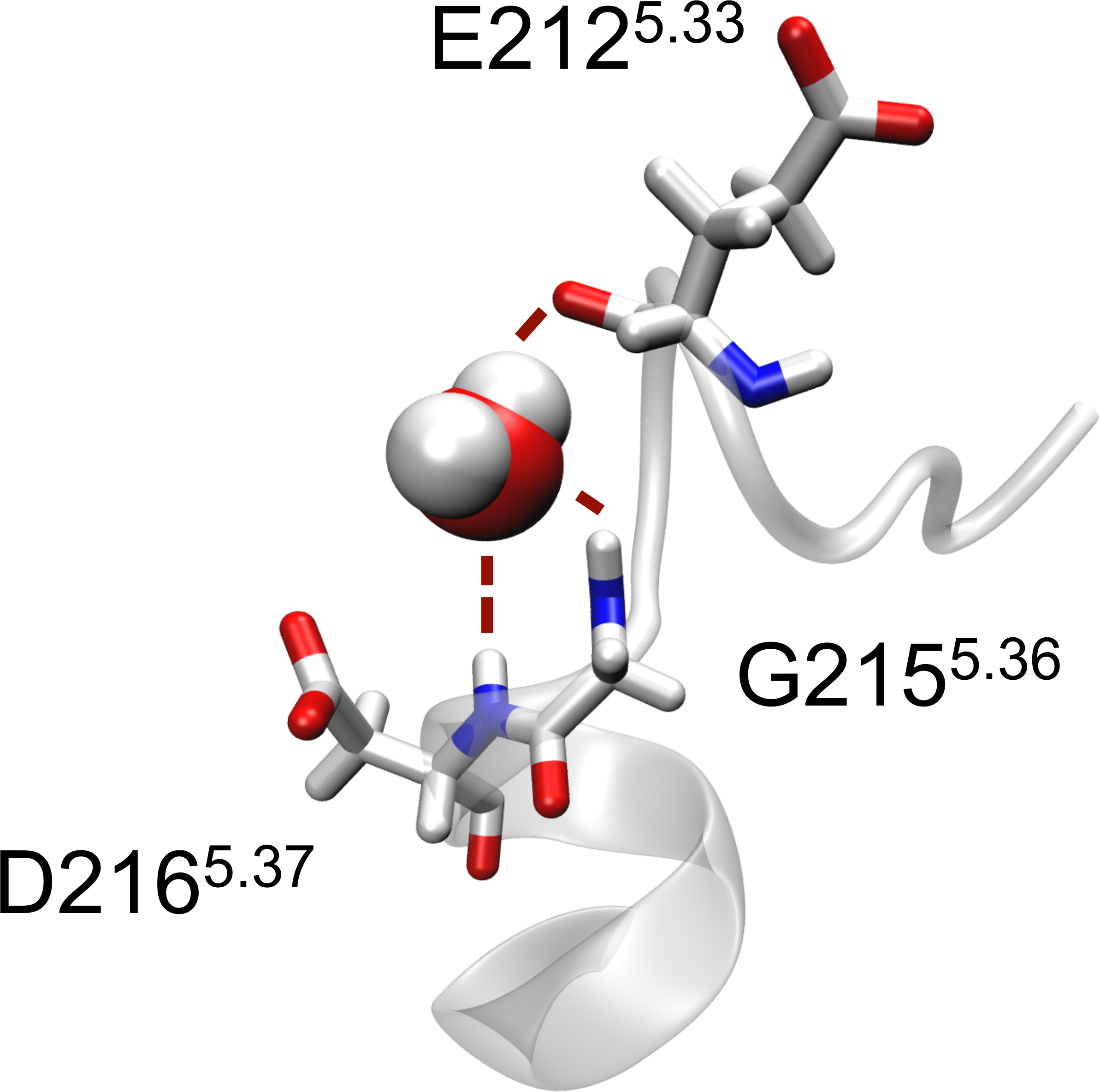

Supplement: Figure S3 — Water-receptor interactions at the 5-HT2BR. Detail of a representative snapshot showing stabilizing interactions between a water molecule and the extracellular part of helix 5. (TIFF) [file pone.0109312.s003.tiff]

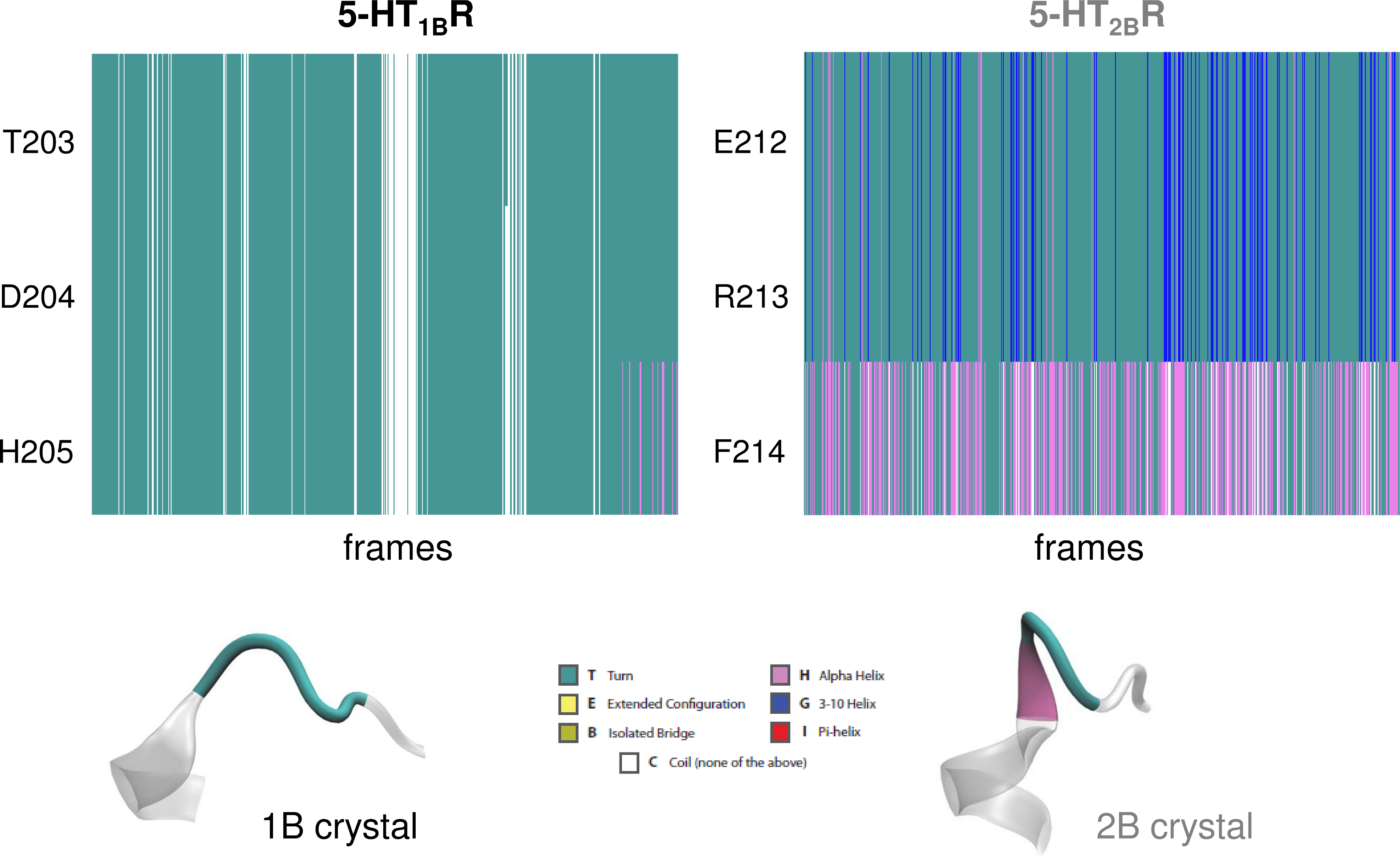

Supplement: Figure S4 — Degree of helix 5 helicity at its extracellular region. This was assessed using the VMD Timeline plugin over the total simulation time of each receptor (2.5 µs). As we can observe, while in the 5-HT1BR this region maintains a coiled secondary structure, in the 5-HT2BR the equivalent residues can adopt a turn conformation corresponding either to an α-helix or to a 3–10 helix. Secondary structure assignment via VMD of the original crystal conformations is provided below each of the plots. (TIFF) [file pone.0109312.s004.tiff]

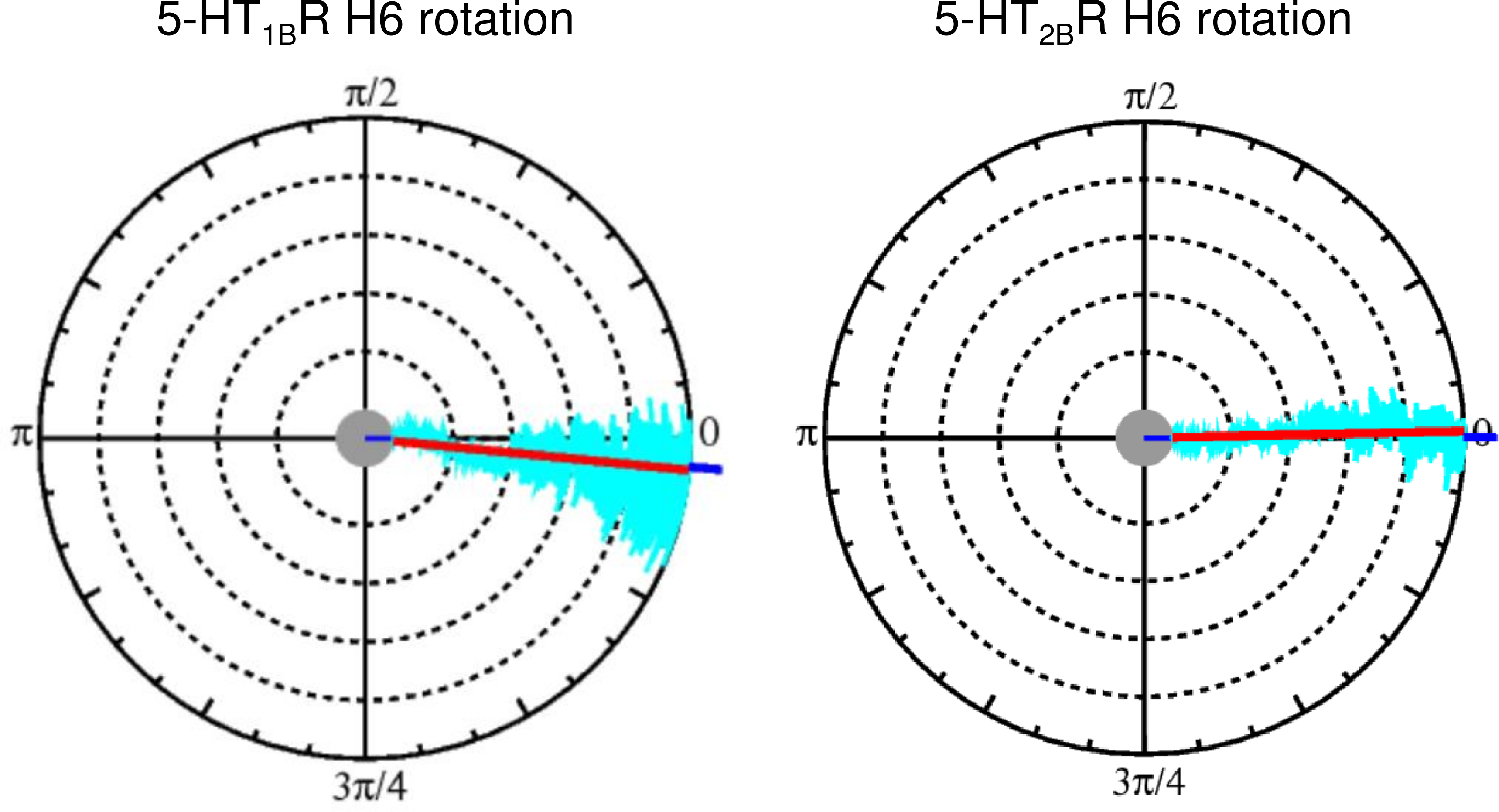

Supplement: Figure S5 — Comparison of the amount of helix 6 rotation considering the extracellular half of the 5-HT1B and 5-HT2B receptors. The amount of helix 6 rotation ranging from position 6.44 to position 6.60 was measured using the Trajelix module of the SIMULAID framework for the analysis of molecular dynamics simulations. This analysis measured rotation around the helix axis perpendicular to the membrane by considering the C alpha residues at each receptor. As we can see, helix 6 is capable of rotating to a higher degree in the 5-HT1BR. (TIFF) [file pone.0109312.s005.tiff]

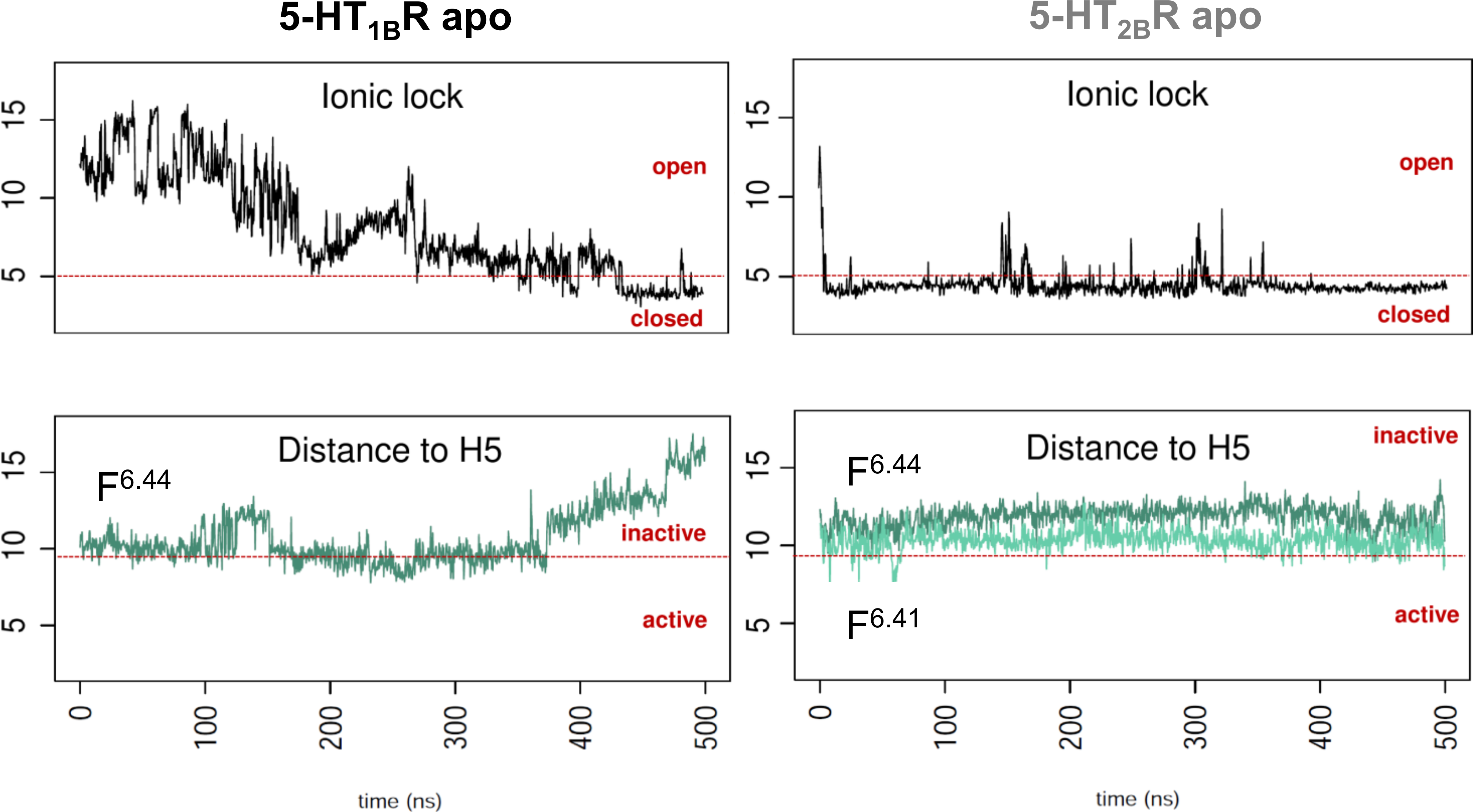

Supplement: Figure S6 — Inactivation of the 5-HT1BR and the 5-HT2BR in the absence of ergotamine. The upper plots monitor distance between residues forming the ionic lock (in particular of Cε of R3.50 and of Cδ of E6.30). The lower plot monitors distance of F6.44 to helix 5 in both receptors (dark green line, measured as distance between C-4 of this residue and Cα of residue 5.50 on H5) and distance to H5 of F6.41 in the 5-HT2BR (light green line, measured as distance between C-4 of this residue and Cα of residue 5.54 on H5). In these plots, we can see how, in both receptors, the ionic lock tends to close in the absence of ergotamine. In the 5-HT2BR, however, this process is much faster than in the 5-HT1BR. Regarding the P-I-F motif, F6.44 adopts an inactivated position from the beginning of the simulation, which is maintained over the 500 ns. (TIFF) [file pone.0109312.s006.tiff]

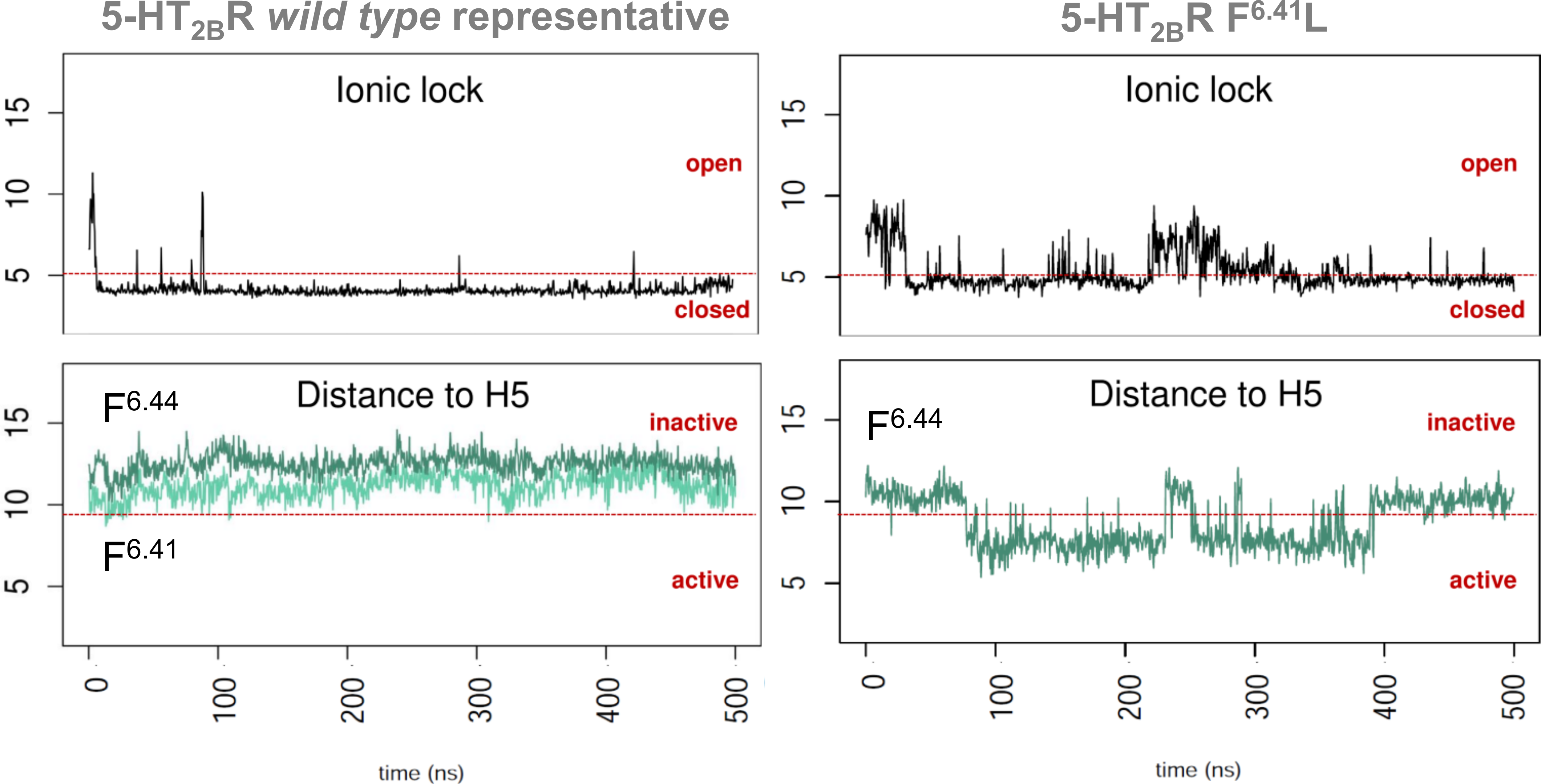

Supplement: Figure S7 — Dynamic behavior of a representative replicate of the wild type 5-HT2BR and of a 5-HT2BR F6.41L mutant. The upper plot monitors distance between residues forming the ionic lock (in particular of Cε of R3.50 and of Cδ of E6.30). The lower plot monitors distance of F6.44 to helix 5 (dark green line, measured as distance between C-4 of this residue and Cα of residue 5.50 on H5). In the case of the wild type receptor distance to H5 of F6.41 in the 5-HT2BR is also monitored (light green line, measured as distance between C-4 of this residue and Cα of residue 5.54 on H5) These plots show how, in this mutant receptor, the ionic lock and residue F6.44 show a bigger tendency to be in their active state. (TIF) [file pone.0109312.s007.tif]

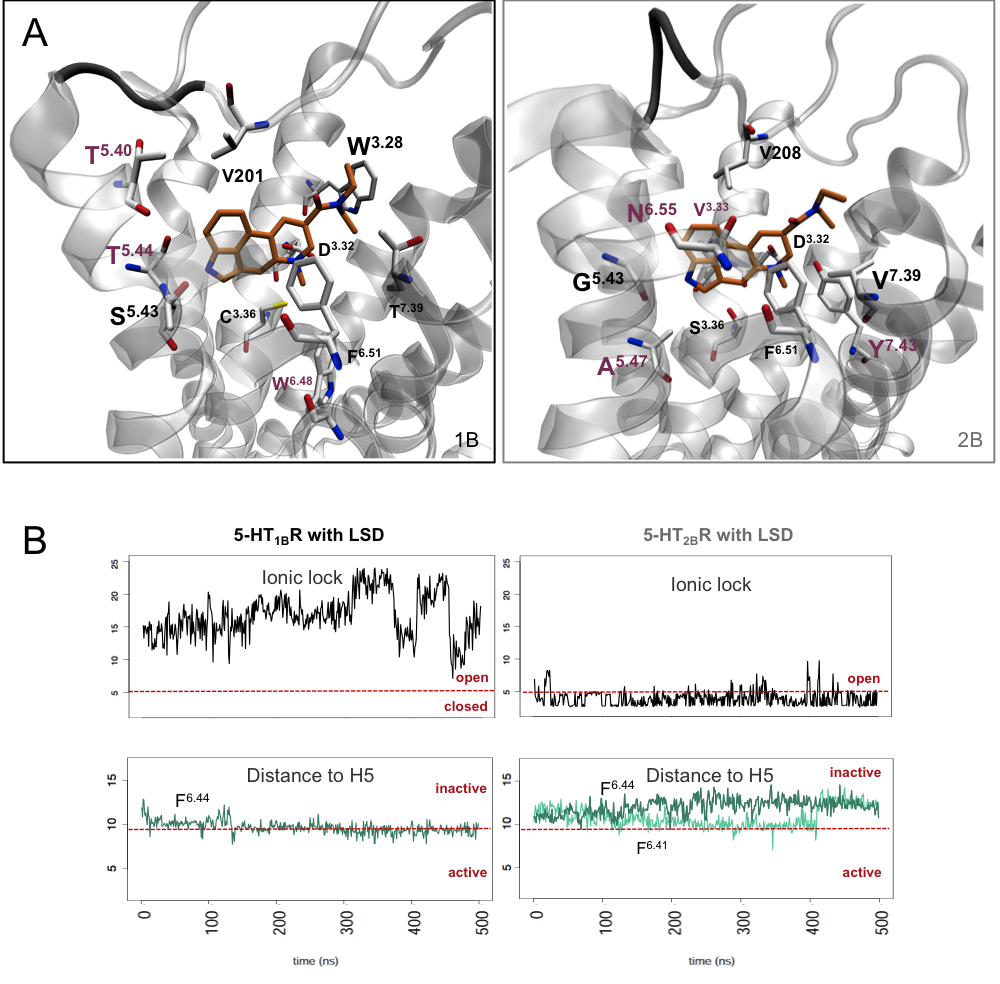

Supplement: Figure S8 — Dynamic behavior of the 5-HT1BR and 5-HT2BR in complex with LSD. A) Analysis of the highest interacting residues with LSD (using a cutoff distance of 3 Å to the ligand). Purple residues correspond to differential ligand-receptor contacts while residues with an increased font represent different interactions as compared to simulations including ergotamine. B) The upper plot monitors distance between residues forming the ionic lock (in particular of Cε of R3.50 and of Cδ of E6.30). The lower plot monitors distance of F6.44 to helix 5 (dark green line, measured as distance between C-4 of this residue and Cα of residue 5.50 on H5). In the case of the 5-HT2BR the distance to H5 of F6.41 is also monitored (light green line, measured as distance between C-4 of this residue and Cα of residue 5.54 on H5). (TIF) [file pone.0109312.s008.tif]

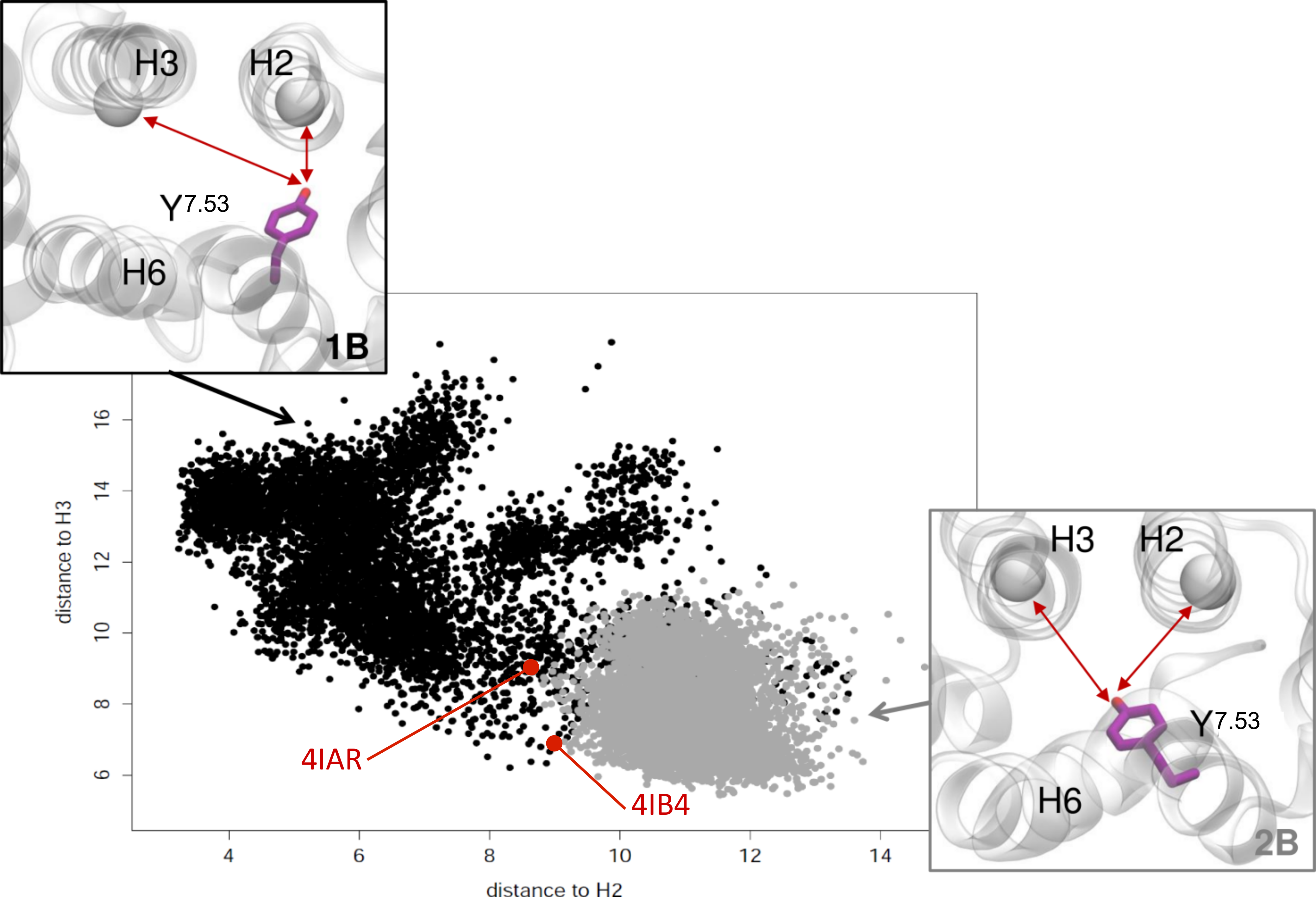

Supplement: Figure S9 — Scatterplot describing the relative distances of Y7.53 of the NPxxY motif to different receptor helices over the simulation replicates of both receptors. As we can see, in the 5-HT2B receptor (grey points), Y7.53 maintains a conformation closer to H3 (distance measured from OH of Y7.53 to Cα of residue 3.50). Conversely, in the 5-HT1BR (black points), this tyrosine adopts a position closer to H2 (reference atom Cα of residue 2.40). Red dots represent the distances observed in the crystal structures of the 5-HT1BR (4IAR) and 5-HT2BR (4IB4). (TIFF) [file pone.0109312.s009.tiff]
